# Supplementary material for: Design, development, and validation of new fluorescent strains for studying oral streptococci
Source: Microbiol Spectr. 2025 Jul 2;13(8):e00168-25. doi: 10.1128/spectrum.00168-25 (PMC12323655; doi:10.1128/spectrum.00168-25)
Supplement: Supplemental material — Fig. S1 to S7; Tables S1 to S4. [file spectrum.00168-25-s0001.pdf]

Supplemental Material

Design, Development and Validation of New Fluorescent Strains for Studying Oral Streptococci

Daniel I. Peters, Iris J. Shin, Alyssa Deever and Justin R. Kaspar [#]

Division of Biosciences, The Ohio State University College of Dentistry, Columbus, Ohio

Table of Contents:

Supplemental Figures (S1-S7): Pages 2 - 8

Supplemental Tables (Table S1-S4): Pages 9 – 14

Supplemental References: Page 15

[#] Corresponding author

Mailing address:
Division of Biosciences, The Ohio State University, College of Dentistry,
305 W. 12th Avenue, Postle Hall Rm 4185, Columbus, OH 43210.
Phone: (614) 292-3373
E-mail: kaspar.17@osu.edu

SUPPLEMENTAL FIGURES AND FIGURE LEGENDS

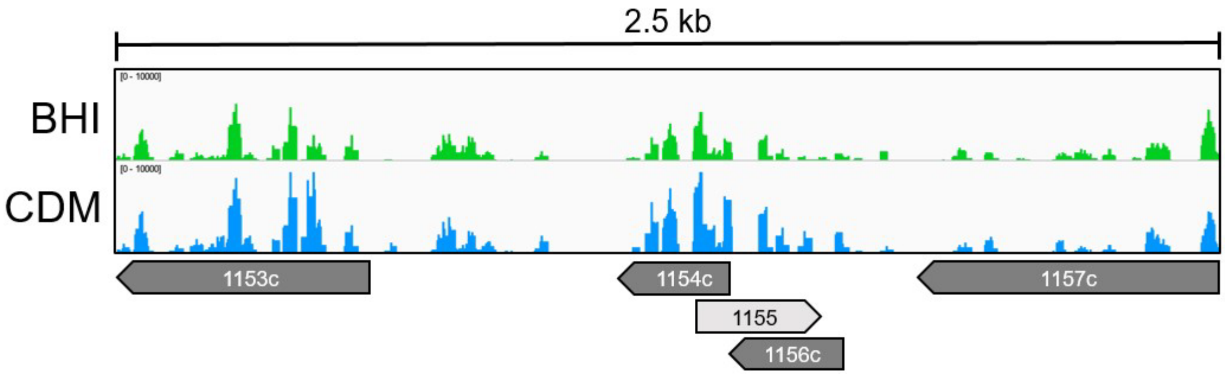

Supplemental Figure 1. Distribution of transposon insertions within SMU.1155. Accumulation of transposon insertions surrounding chosen chromosomal site to insert fluorescent gene fragments in *S. mutans*. Each colored line/peak represents a single transposon sequencing read, and thus insertion of the Magellan6 transposon into this region. Tn-Seq reads from *S. mutans* growth in two conditions were mapped and visualized – growth in BHI (green track) and growth in CDM (blue track). The gene that corresponds to the insertion site is shown in light gray, while surrounding genes are shown in dark gray and indicated by their gene number. Visualization of transposon insertions helps confirm there is no loss of fitness encountered by disruption of SMU.1155. Sequencing data was accessed from Shields, Zeng, Culp and Burne 2018, doi: 10.1125/msphere.00031-18.

cagcaaagaatggcggaaacgtaaaagaagttatggaaataagacttagaagcaaacttaagagtgtgtt
gacagtgcagtaccttaaaattttgtataataggaattgaagttaaattagatgctaaaatttt**GGATCC**
aagaaggagtgattacGAGCTCTAGATCGAATTCCTTATTAACGTTGATATAATTTAAATTTTATTTGAC
AAAAATGGGCTCGTGTTGTACAATAAATGTGATTAACTAATAAGGAGGACAAACatgagcgagctgatta
aggagaacatgcacatgaagctgtacatggagggcaccgtggacaaccatcacttcaagtgcacatccga
gggcgaaggcaagccctacgagggcacccagaccatgagaatcaaggtggtcgagggcggccctctcccc
ttcgccttcgacatcctggctactagcttcctctacggcagcaagaccttcatcaaccacacccagggca
tccccgacttcttcaagcagtccttccctgagggcttcacatgggagagagtcaccacatacgaagacgg
gggcgtgctgaccgctacccaggacaccagcctccaggacggctgcctcatctacaacgtcaagatcaga
ggggtgaacttcacatccaacggccctgtgatgcagaagaaaacactcggctgggaggccttcaccgaga
cgctgtaccccgctgacggcggcctggaaggcagaaacgacatggccctgaagctcgtgggcggagcca
tctgatcgcaaacgccaagaccacatatagatccaagaaacccgctaagaacctcaagatgcctggcgtc
tactatgtggactacagactggaaagaatcaaggaggccaacaacgagacctacgtcgagcagcacgagg
tggcagtggccagatactgcgacctccctagcaaactggggcacaagcttaattaaaacgtaaaagaagt
taatgaggaggatatatttgaatacatacgaacaaattaataaagtgaaaaaaatacttcggaaacattt
aaaaaataaccttattggtacttacatgtttggatcaggagttgagagtggactaaaaccaaatagtgat
cttgacttttttagtcgtcgtatctgaaccattgacagatcaaagtaaagaaatacttatacaaaaaatta
gacctatttcaaaaaaaataggagataaaagcaacttacgatatattgaattaacaattattattcagca
agaaatggtaccgtggaatcatcctcccaaacaagaatttatttatggagaatggttacaagagctttat
gaacaaggatacattcctcagaaggaattaaattcagatttaaccataatgctttaccaagcaaaacgaa
aaaataaaagaatatacggaaattatgacttagaggaattactacctgatattccattttctgatgtgag
aagagccattatggattcgtcagaggaattaatagataattatcaggatgatgaaaccaactctatatta
actttatgccgtatgattttaactatggacacgggtaaaatcataccaaaagatattgcgggaaatgcag
tggctgaatcttctccattagaacatagggagagaattttgttagcagttcgtagttatcttggagagaa
tattgaatggactaatgaaaatgtaaatttaactataaactatttaaataacagattaaaaaaattataa
taaCTCGGTACCAAATTCCAGAAAAGAGGCCTCCCGAAAGGGGGGCCTTTTTTCGTTTTGGTCCttctat
GGATCCcttttgtaaatttggaaagttacacgttactaaagggactgtagatccagcaggtatactactg
acagc

Supplemental Figure 2. Overview of P*veg*:*mtagbfp2* gene fragment design. Sequence of the P*veg*:*mtagbfp2* gene fragment that was synthesized and used in cloning for all mTagBFP2 strains. Several features are either highlighted or in colored text: location of the genefrag-amplify primers is shown in yellow highlight, the BamHI restriction sites are shown in **turquoise highlight and bolded**, the Pveg promoter is shown in PINK CAPITAL letters, the *mtagbfp2* gene is shown in blue letters, *aad9* sequence is shown in light gray letters, and the transcriptional terminator, L3S2P21, is shown in PURPLE CAPITAL letters. The gene fragment was synthesized by Integrated DNA Technologies (IDT).

cagcaaagaatggcggaaacgtaaaagaagttatggaaataagacttagaagcaaacttaagagtgtgtt
gacagtgcagtaccttaaaattttgtataataggaattgaagttaaattagatgctaaaatttt**GGATCC**
aagaaggagtgattacGAGCTCTAGATCGAATTCCTTATTAACGTTGATATAATTTAAATTTTATTTGAC
AAAAATGGGCTCGTGTTGTACAATAAATGTGATTAACTAATAAGGAGGACAAACatgtcaaaggagaag
agctgttcacaggtgttgtgccgattctcgttgagcttgacggagatgtaaacggacacaaattctctgt
tcgcggtgaaggtgaaggagatgcaacaaacggcaagctgacattgaagtttatttgcacaactggaaag
ctgccggttccttggccgacacttgtaacgacgctgacttacggcgttcaatgcttctctcgttatccag
accacatgaaacgccatgatttcttcaaatctgcaatgcctgaaggctacgttcaagagcgtacgatcag
cttcaaagatgacggaacgtacaaaacaagagcagaagtgaagtttgaaggtgacacacttgtgaaccgc
attgaattgaaaggcattgatttcaaagaagatggaaacatccttggacacaaacttgaatacaacttca
acagccacaacgtatacatcactgctgacaaacaaaaaaacggcatcaaagcaaacttcaaaatccgtca
taacgtagaggacggttctgttcagcttgctgatcattatcagcaaaatacaccgatcggtgacggcccg
gttcttcttcctgataaccattatttatcaactcaaagcgtattatcaaaagacccaaatgaaaagcgtg
accacatggtgctgcttgaatttgtgacagctgctggtatcactcacggcatggatgagctttataagta
atttggaaagttacacgatgagacgcatttaccttaatacatatgagcagatcaacaaggtgaagaagat
tttaagaaagcacttaaaaaataatcttattggcacgtatatgttcggaagcggtgtcgaatcaggtctt
aagccgaattctgacttagacttcttggtcgttgtctctgagcctttaacggaccaatctaaagaaattt
tgattcaaaaaattcgccctatctcaaagaaaatcggtgacaagtcaaatttgagatacattgaattaac
catcatcatccagcaagaaatggtcccgtggaaccacccgccgaagcaagagttcatttacggcgaatgg
ttacaggagttgtatgagcaaggctacattccacagaaagagcttaatagtgacttgacaatcatgttat
atcaggcaaaacgtaagaacaaacgcatttacggaaactatgatttagaggaacttttgcccgatatccc
attttctgacgttcgtcgcgccattatggacagctctgaggagttaattgataactaccaggatgatgag
acgaatagtattttaactctttgtcgtatgattttgacaatggacactggtaaaatcatccccaaggata
ttgctggtaatgccgttgcagaaagcagcccattggagcacagagagcgcattcttcttgcagtacgcag
ctatcttggagagaatatcgagtggacaaacgagaatgttaatttgacaatcaattatttgaacaatcgc
ttgaaaaagctttaataaCTCGGTACCAAATTCCAGAAAAGAGGCCTCCCGAAAGGGGGGCCTTTTTTCG
TTTTGGTCCttctat**GGATCC**cttttgtaaatttggaaagttacacgttactaaagggactgtagatcca
gcaggtatactactgacagc

Supplemental Figure 3. Overview of P*veg*:*sfgfp* gene fragment design. Sequence of the P*veg*:*sfgfp* gene fragment that was synthesized and used in cloning for all sfGFP strains. Several features are either highlighted or in colored text: location of the 'genefrag-amplify' primers is shown in yellow highlight, the BamHI restriction sites are shown in **turquoise highlight and bolded**, the Pveg promoter is shown in PINK CAPITAL letters, the *sfgfp* gene is shown in green letters, *aad9* sequence** is shown in light gray letters, and the transcriptional terminator, L3S2P21, is shown in PURPLE CAPITAL letters. The gene fragment was synthesized by Integrated DNA Technologies (IDT).

**Note: the *aad9* sequence in this gene fragment underwent codon optimization for *S. mutans* using IDT's codon optimizer tool. This was done in order to meet the gene fragment complexity benchmarks during IDT gene block order submission (synthesis of a gene block using the *aad9* sequence included in the other two fragments failed and was re-attempted and successful with this sequence).

cagcaaagaatggcggaaacgtaaaagaagttatggaaataagacttagaagcaaacttaagagtgtgtt
gacagtgcagtaccttaaaattttgtataataggaattgaagttaaattagatgctaaaaatttt**GGATCC**
aagaaggagtgattacGAGCTCTAGATCGAATTCCTTATTAACGTTGATATAATTTAAATTTTATTTGAC
AAAAATGGGCTCGTGTTGTACAATAAATGTGATTAACTAATAAGGAGGACAAACatggattctaccgaag
ctgttatcaaagagttcatgcgttttaaggtacacatggagggctcaatgaatggacacgaatttgaaat
tgaaggagagggtgagggtcgcccgtacgaaggcacgcaaacggctaaattgaaagtaacgaaaggcggc
ccgttgccatttagttgggacatcttgtcaccgcaatttatgtatggttcacgcgcttttatcaagcacc
cggccgatattcctgactactggaagcaatcattcccagagggcttcaagtgggagcgcgttatgatctt
cgaggacggtggcacagtctcagttacgcaagacacctctcttgaggacggaactttgatttacaaagtg
aagttgcgtggaggtaatttcccgcccgatggaccggtcatgcagaaacgtaccatgggctgggaggctt
caacagagcgcctttacccggaagatgtcgtacttaaaggcgacatcaagatggcattgcgtttgaaaga
tggtggacgttaccttgccgatttcaagaccacctataaggcaaaaaagccagtgcaaatgcccggcgcc
tttaatattgaccgcaagttggacatcacgagtcacaatgaggactatactgttgttgagcaatatgagc
gttctgttgcaagacattcaacaggtggtagcggcggtagttaagagtgtgttgacagtgatgaggagga
tatatttgaatacatacgaacaaattaataaagtgaaaaaaatacttcggaaacatttaaaaaataacct
tattggtacttacatgtttggatcaggagttgagagtggactaaaaccaaatagtgatcttgacttttta
gtcgtcgtatctgaaccattgacagatcaaagtaaagaaatacttatacaaaaaattagacctatttcaa
aaaaaataggagataaaagcaacttacgatatattgaattaacaattattattcagcaagaaatggtacc
gtggaatcatcctcccaaacaagaatttatttatggagaatggttacaagagctttatgaacaaggatac
attcctcagaaggaattaaattcagatttaaccataatgctttaccaagcaaaacgaaaaaataaaagaa
tatacggaaattatgacttagaggaattactacctgatattccattttctgatgtgagaagagccattat
ggattcgtcagaggaattaatagataattatcaggatgatgaaaccaactctatattaactttatgccgt
atgattttaactatggacacgggtaaaatcataccaaaagatattgcgggaaatgcagtggctgaatctt
ctccattagaacatagggagagaattttgttagcagttcgtagttatcttggagagaatattgaatggac
taatgaaaatgtaaatttaactataaactatttaaataacagattaaaaaattataataaCTCGGTACC
AAATTCCAGAAAAGAGGCCTCCCGAAAGGGGGGCCTTTTTTCGTTTTGGTCCttctat**GGATCC**ctttg
taaatttggaaagttacacgttactaaagggactgtagatccagcaggtatactactgacagc

Supplemental Figure 4. Overview of P*veg*:*mscarlet-I3* gene fragment design. Sequence of the P*veg*:*mscarlet-I3* gene fragment that was synthesized and used in cloning for all mScarlet-I3 strains. Several features are either highlighted or in colored text: location of the 'genefrag-amplify' primers is shown in yellow highlight, the BamHI restriction sites are shown in **turquoise highlight and bolded**, the Pveg promoter is shown in PINK CAPITAL letters, the *mscarlet-I3* gene is shown in red letters, *aad9* sequence is shown in light gray letters, and the transcriptional terminator, L3S2P21, is shown in PURPLE CAPITAL letters. The gene fragment was synthesized by Integrated DNA Technologies (IDT).

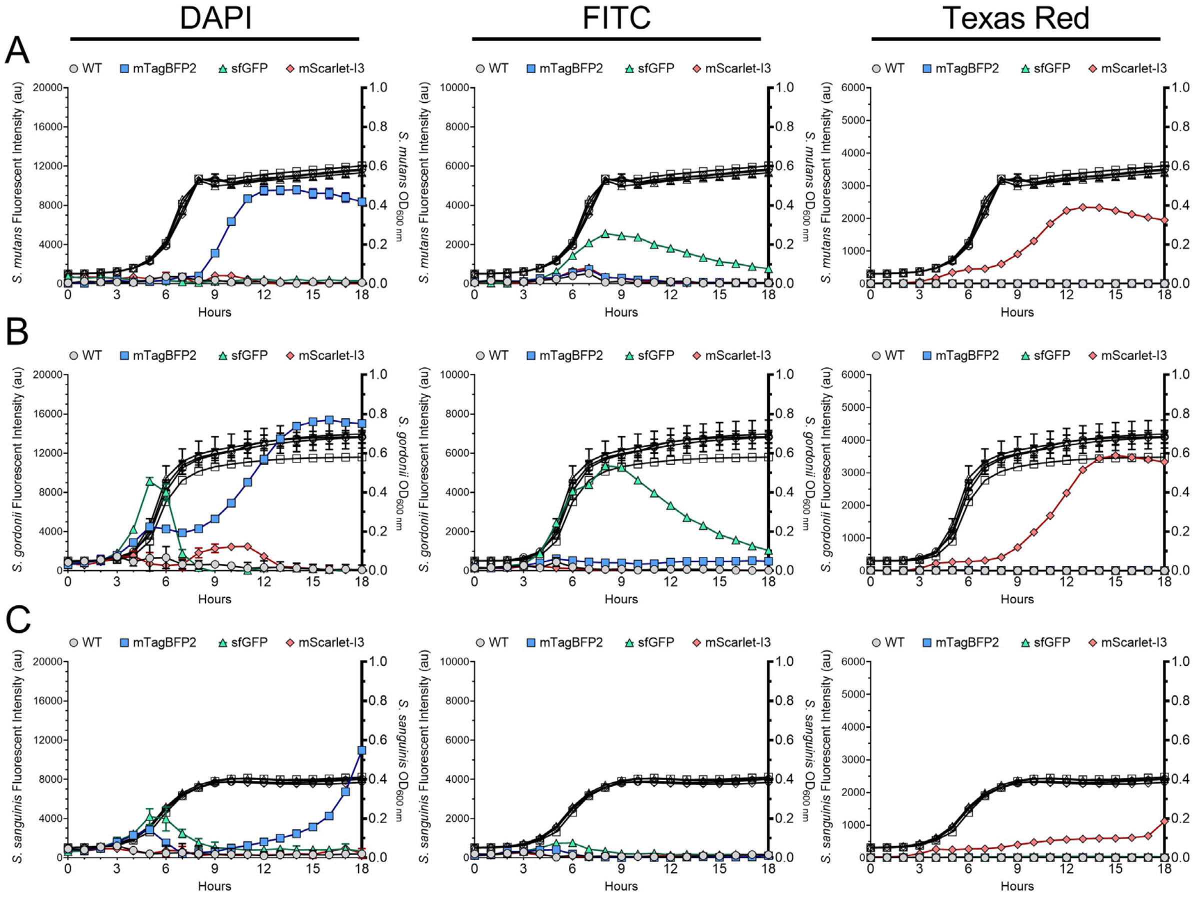

Supplemental Figure 5. Growth rates of fluorescent strains overlayed with fluorescent intensity production. Relative fluorescent intensity (arbitrary units, a.u., left y-axis) over 18 h for either the wild-type (WT, gray circles) strain or fluorescent strains mTagBFP2 (blue squares), sfGFP (green triangles) and mScarlet-I3 (red diamonds) in either **(A)** *S. mutans*, **(B)** *S. gordonii* or **(C)** *S. sanguinis*. The left column represents intensities in the DAPI channel (Ex 399 / Em 455), the middle column represents the FITC channel (Ex 485 / Em 528), and the right column the Texas Red channel (Ex 550 / Em 590). This is the same data as in Figure 3, but with growth curves of each strain also included and corresponds to optical density at 600 nm values displayed on the right y-axis. Growth curves of each strain is represented by their same symbol listed above, but in black and white.

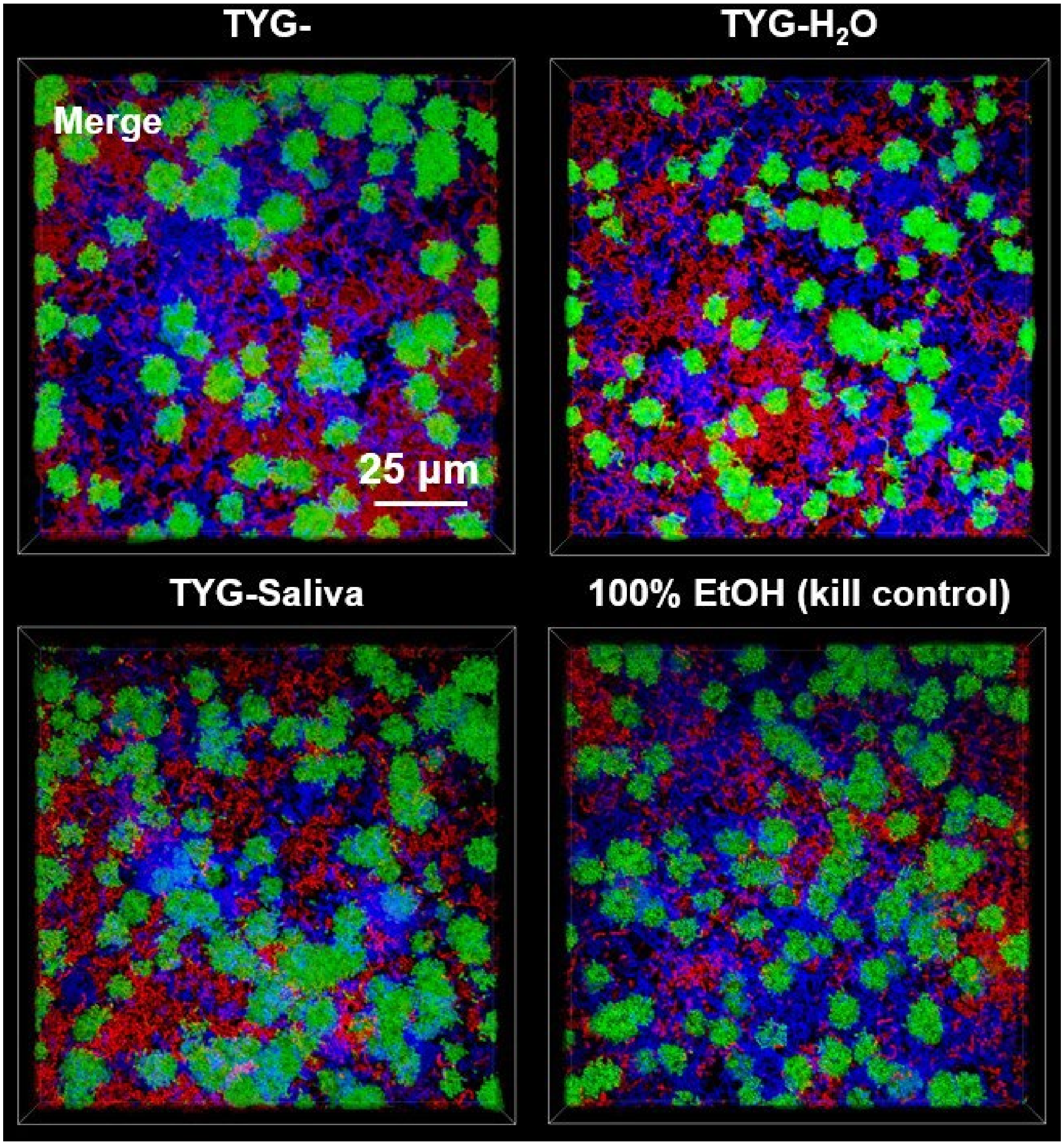

Supplemental Figure 6. Merged biofilm images from Figure 6 without the SYTOX Red channel overlay. Maximum intensity, 100x 3D models of a super-resolution confocal-captured biofilm image oriented from the top down (Z+) of *S. sanguinis* (mTagBFP2, blue), *S. mutans* (sfGFP, green), and *S. gordonii* (mScarlet-I3, red) tricultures grown in TYG-, TYG-H_2O, TYG-Saliva or TYG- with 100% ethanol (EtOH) applied for 15 minutes as a kill control. Biofilms were grown for 24 h with 5 mM sucrose prior to imaging. These are the same merged images from Figure 6, but without the SYTOX Red (CY5 filter) overlay. Scale bar (25 µm) is shown in the top left merged image. Images are 127 µm (L) x 127 µm (W) x 30 µm (H).

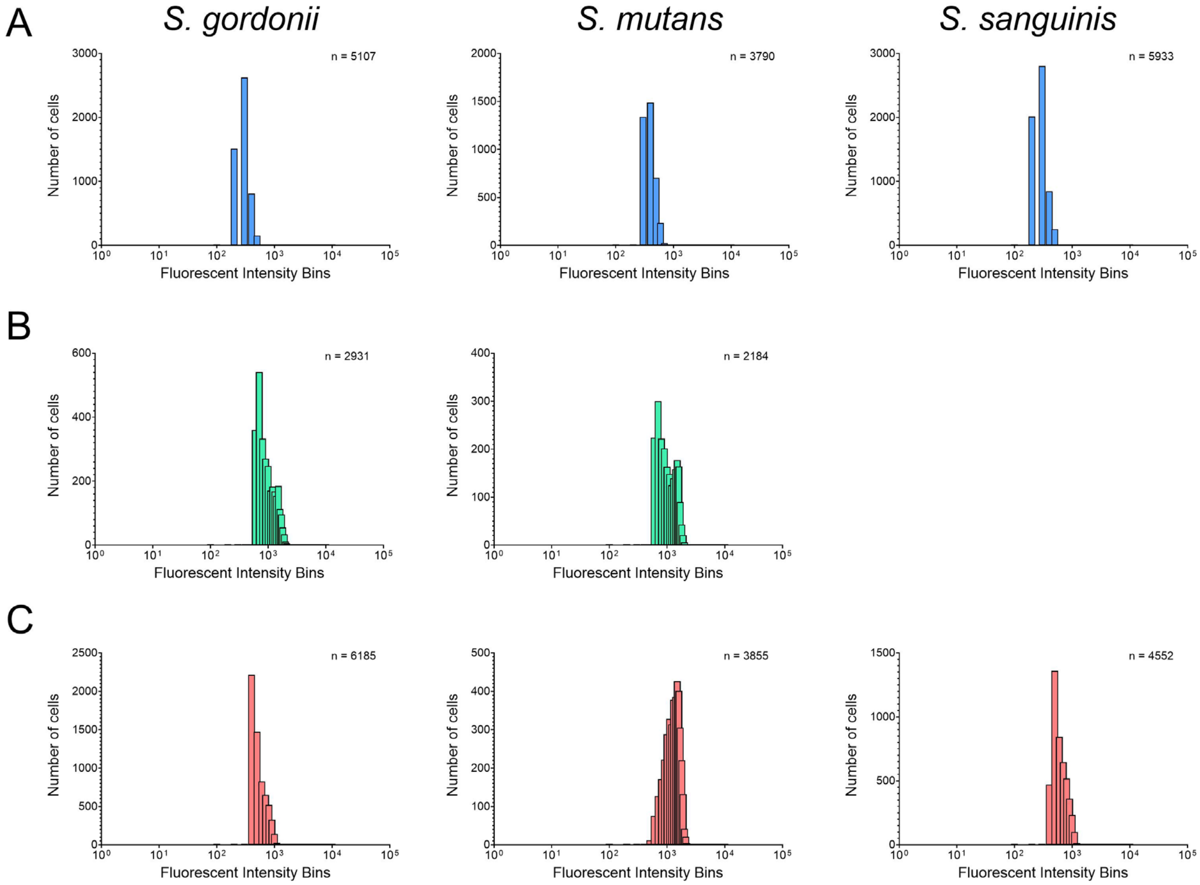

Supplemental Figure 7. Recorded fluorescent intensity ranges of each strain on a single-cell basis. Histograms of binned relative fluorescent intensity (a.u.) within individual cells using single-cell analysis from fluorescent strains growin in monoculture biofilms expressing either **(A)** mTagBFP2, **(B)** sfGFP or **(C)** mScarlet-I3. Each histogram represents the fluorescent intensity from either (left to right) *S. gordonii*, *S. mutans* or *S. sanguinis*. The height of each bar represents the number of cells (y-axis) within each fluorescent intensity bin. The number of cells (n) analyzed for each graph is shown in the upper right corner.

SUPPLEMENTAL TABLES

Supplemental Table 1. Bacterial strains used in this study.

Species	Strain	Genotype or description	Antibiotic resistance	Reference or source
Streptococcus gordonii	DL1			Lab Stock
	Δ2075	Allelic exchange of SGO_2075, a gene not transcriptionally active in TYG, with *ermB* providing resistance to erythromycin	Erythromycin	This manuscript
	Δ2075::P*veg*:*mtagbfp*2	Allelic exchange of SGO_2075 with a gene block containing the fluorescent gene *mtagbfp2*, driven by the P*veg* promoter and carrying *aad9* for spectinomycin resistance	Spectinomycin	This manuscript
	Δ2075::P*veg*:*sfgfp*	Allelic exchange of SGO_2075 with a gene block containing the fluorescent gene *sfgfp*, driven by the P*veg* promoter and carrying *aad9* for spectinomycin resistance	Spectinomycin	This manuscript
	Δ2075::P*veg*:*mscarlet-I3*	Allelic exchange of SGO_2075 with a gene block containing the fluorescent gene *mscarlet-I3*, driven by the P*veg* promoter and carrying *aad9* for spectinomycin resistance	Spectinomycin	This manuscript
Streptococcus mutans	UA159			Lab Stock
	pMZ- / UA159	UA159 with pMZ plasmid integrated into genome. Serves as a "marked" *S. mutans* strain during colony forming unit (CFU) assays	Kanamycin	(Shields et al. 2019)
	Δ1155	Allelic exchange of SMU_1155, a gene not transcriptionally active in TYG, with *ermB* providing resistance to erythromycin	Erythromycin	This manuscript

	Δ1155::P*veg*:*mtagbfp*2	Allelic exchange of SMU_1155 with a gene block containing the fluorescent gene *mtagbfp2*, driven by the P*veg* promoter and carrying *aad9* for spectinomycin resistance	Spectinomycin	This manuscript
	Δ1155::P*veg*:*sfgfp*	Allelic exchange of SMU_1155 with a gene block containing the fluorescent gene *sfgfp*, driven by the P*veg* promoter and carrying *aad9* for spectinomycin resistance	Spectinomycin	This manuscript
	Δ1155::P*veg*:*mscarlet-I3*	Allelic exchange of SMU_1155 with a gene block containing the fluorescent gene *mscarlet-I3*, driven by the P*veg* promoter and carrying *aad9* for spectinomycin resistance	Spectinomycin	This manuscript
Streptococcus sanguinis	SK36			Lab Stock
	Δ2030	Allelic exchange of SSA_2030, a gene not transcriptionally active in TYG, with ermB providing resistance to erythromycin	Erythromycin	This manuscript
	Δ2030::P*veg*:*mtagbfp*2	Allelic exchange of SSA_2030 with a gene block containing the fluorescent gene *mtagBFP2*, driven by the P*veg* promoter and carrying *aad9* for spectinomycin resistance	Spectinomycin	This manuscript
	Δ2030::Pveg:*sfgfp*	Allelic exchange of SSA_2030 with a gene block containing the fluorescent gene *sfgfp*, driven by the P*veg* promoter and carrying *aad9* for spectinomycin resistance	Spectinomycin	This manuscript
	Δ2030::Pveg:*mscarlet-I3*	Allelic exchange of SSA_2030 with a gene block containing the fluorescent gene *mscarlet-I3*, driven by the P*veg* promoter and carrying *aad9* for spectinomycin resistance	Spectinomycin	This manuscript

Supplemental Table 2. Primers used in this study.

Species and Strain	Primer Name	Primer Sequence (5' - 3')*	Tm
	genefrag-amplify-F	GAG TGT GTT GAC AGT GCA GTA CC	58
	genefrag-amplify-R	CCT GCT GGA TCT ACA GTC C	55
	genefrag-check-F	GTT TGG ATC AGG AGT TGA GAG TGG	57
	genefrag-check-R	CTA ATG GAG AAG ATT CAG CCA CTG C	58
Streptococcus gordonii DL1	SGO_2075_A	TTC CTA TAG GAA TAG GAA CAG GGC	55
	SGO_2075_B	CTA <u>**GGA TCC**</u> TTT CGG GTG CAC TTT CCT G	62
	SGO_2075_C	TCT <u>**GGA TCC**</u> ACG TCG GTA TAA CCC	59
	SGO_2075_D	CTT AGC CGG GTT AGT AGT CAA GCC	59
Streptococcus mutans UA159	SMU_1155_A	CTC ATA GGA ATC AAC TGG ACA GGC C	59
	SMU_1155_B	GAT <u>**GGA TCC**</u> CGG GAT CGT CTG GCC	65
	SMU_1155_C	AAG <u>**GGA TCC**</u> CGC AGC TCT TCC ACC	65
	SMU_1155_D	TTC CAC AGT GAC CTC AGA AGC TGC	61
Streptococcus sanguinis SK36	SSA_2030_A	GTC TTC TTG GAC TTG CAG AGC TGC	60
	SSA_2030_B	GGT <u>**GGA TCC**</u> TGC TTG TTC ATA GCT TGG CG	64
	SSA_2030_C	CAT <u>**GGA TCC**</u> ATT ATC GGC TAT CCC AG	58
	SSA_2030_D	TAA GTA ACG TTC CAG AGC CTC CTC	57

*<u>**Bold and underline**</u> denotes BamHI cut site used in the PCR ligation mutagenesis approach

The ‘genefrag-amplify’ primers were used to amplify the entire fluorescent gene fragment synthesized by IDT prior to BamHI restriction digest.

The ‘genefrag-check’ primers were used during colony PCR to screen for desired transformants after transformation of the linear ligation product. These primers amplify a region within the *aad9* gene.

Supplemental Table 3. Sequences and sources of individual components of the fluorescent gene fragments.

Component	Sequence	Source
P*veg* promoter	gagctctagatcgaattccttattaacgttgatataatttaaattttatttgacaaaaatgggctcgtg<br>ttgtacaataaatgtgattaactaataaggaggacaaac	(Shields et al. 2019)
aad9 gene	atgaggaggatatatttgaatacatacgaacaaattaataaagtgaaaaaatacttcggaaa<br>catttaaaaaataaccttattggtacttacatgtttggatcaggagttgagagtggactaaaacca<br>aatagtgatcttgactttttagtcgtcgtatctgaaccattgacagatcaaagtaaagaaatacttat<br>acaaaaaattagacctatttcaaaaaaaataggagataaaagcaacttacgatatattgaatta<br>acaattattattcagcaagaaatggtaccgtggaatcatcctcccaaacaagaatttatttatgga<br>gaatggttacaagagctttatgaacaaggatacattcctcagaaggaattaaattcagatttaac<br>cataatgctttaccaagcaaaacgaaaaaataaaagaatatacggaaattatgacttagagg<br>aattactacctgatattccattttctgatgtgagaagagccattatggattcgtcagaggaattaata<br>gataattatcaggatgatgaaaccaactctatattaactttatgccgtatgattttaactatggacac<br>gggtaaaatcataccaaaagatattgcgggaaatgcagtggctgaatcttctccattagaacat<br>agggagagaattttgttagcagttcgtagttatcttggagagaatattgaatggactaatgaaaat<br>gtaaatttaactataaactatttaaataacagattaaaaaaattataataa	(Benson and Haldenwang 1993; Guérout-Fleury et al. 1995; LeDeaux and Grossman 1995)
L3S2P21 transcriptional terminator	ctcggtaccaaattccagaaaagaggcctcccgaaagggggcctttttcgttttggtcc	(Chen et al. 2013)
mtagbfp2 gene	atgagcgagctgattaaggagaacatgcacatgaagctgtacatggagggcaccgtggaca<br>accatcacttcaagtgcacatccgagggcgaaggcaagccctacgagggcacccagaccat<br>gagaatcaaggtggtcgagggcggccctctcccсttcgccttcgacatcctggctactagcttcc<br>tctacggcagcaagaccttcatcaaccacacccagggcatccccgacttcttcaagcagtcctt<br>ccctgagggcttcacatgggagagagtcaccacatacgaagacgggggcgtgctgaccgct<br>acccaggacaccagcctccaggacggctgcctcatctacaacgtcaagatcagaggggtga<br>acttcacatccaacggccctgtgatgcagaagaaaacactcggctgggaggccttcaccgag<br>acgctgtaccccgctgacggcggcctggaaggcagaaacgacatggccctgaagctcgtgg<br>gcgggagccatctgatcgcaaacgccaagaccacatatagatccaagaaacccgctaaga<br>acctcaagatgcctggcgtctactatgtggactacagactggaaagaatcaaggaggccaac<br>aacgagacctacgtcgagcagcacgaggtggcagtggccagatactgcgacctccctagca<br>aactggggcacaagcttaattaa	(Subach et al. 2011)
sfgfp gene	atgtcaaaaggagaagagctgttcacaggtgttgtgccgattctcgttgagcttgacggagatgt<br>aaacggacacaaattctctgttcgcggtgaaggtgaaggagatgcaacaaacggcaagctg<br>acattgaagtttatttgcacaactggaaagctgccggttccttggccgacacttgtaacgacgctg<br>acttacggcgttcaatgcttctctcgttatccagaccacatgaaacgccatgatttcttcaaatctgc<br>aatgcctgaaggctacgttcaagagcgtacgatcagcttcaaagatgacggaacgtacaaaa<br>caagagcagaagtgaagtttgaaggtgacacacttgtgaaccgcattgaattgaaaggcattg<br>atttcaaagaagatggaaacatccttggacacaaacttgaatacaacttcaacagccacaacg<br>tatacatcactgctgacaaacaaaaaaacggcatcaaagcaaacttcaaaatccgtcataac<br>gtagaggacggttctgttcagcttgctgatcattatcagcaaaatacaccgatcggtgacggccc<br>ggttcttcttcctgataaccattatttatcaactcaaagcgtattatcaaaagacccaaatgaaaa<br>gcgtgaccacatggtgctgcttgaatttgtgacagctgctggtatcactcacggcatggatgagct<br>ttataagtaa	(Overkamp et al. 2013)

mscarlet-I3 gene	atggattctaccgaagctgttatcaaagagttcatgcgttttaaggtacacatggagggctcaatg<br>aatggacacgaatttgaaattgaaggagagggtgagggtcgcccgtacgaaggcacgcaaa<br>cggctaaattgaaagtaacgaaaggcggcccgttgccatttagttgggacatcttgtcaccgca<br>atttatgtatggttcacgcgcttttatcaagcacccggccgatattcctgactactggaagcaatca<br>ttcccagagggcttcaagtgggagcgcgttatgatcttcgaggacggtggcacagtctcagttac<br>gcaagacacctctcttgaggacggaactttgatttacaaagtgaagttgcgtggaggtaatttcc<br>cgcccgatggaccggtcatgcagaaacgtaccatgggctgggaggcttcaacagagcgcctt<br>tacccggaagatgtcgtacttaaaggcgacatcaagatggcattgcgtttgaaagatggtggac<br>gttaccttgccgatttcaagaccacctataaggcaaaaaagccagtgcaaatgcccggcgcct<br>ttaatattgaccgcaagttggacatcacgagtcacaatgaggactatactgttgttgagcaatatg<br>agcgttctgttgcaagacattcaacaggtggtagcggcggtagttaa	(Gadella et al. 2023)

Supplemental Table 4. Concentration of inoculated bacterial strains for competitive index and biofilm triculture experiments.

from (Choi et al. 2024)

Strain Name	Concentration of Competitor	*S. mutans* Concentration
S. gordonii DL1	10% solution (1:10 dilution); 1x	1x
S. sanguinis SK36	1x	1x

REFERENCES

Benson AK, Haldenwang WG. 1993. Regulation of σ(B) levels and activity in *Bacillus subtilis*. J Bacteriol. 175(8):2347–2356.

Chen YJ, Liu P, Nielsen AAK, Brophy JAN, Clancy K, Peterson T, Voigt CA. 2013. Characterization of 582 natural and synthetic terminators and quantification of their design constraints. Nat Methods. 10(7):659–664.

Choi A, Dong K, Williams E, Pia L, Batagower J, Bending P, Shin I, Peters DI, Kaspar JR. 2024. Human saliva modifies growth, biofilm architecture, and competitve behaviors of oral streptococci. mSphere. 9(2):e00771-23.

Gadella TWJ, van Weeren L, Stouthamer J, Hink MA, Wolters AHG, Giepmans BNG, Aumonier S, Dupuy J, Royant A. 2023. mScarlet3: a brilliant and fast-maturing red fluorescent protein. Nat Methods. 20(4):541–545.

Guérout-Fleury AM, Shazand K, Frandsen N, Stragier P. 1995. Antibiotic-resistance cassettes for *Bacillus subtilis*. Gene. 167(1–2):335–336.

LeDeaux JR, Grossman AD. 1995. Isolation and characterization of *kinC*, a gene that encodes a sensor kinase homologous to the sporulation sensor kinases KinA and KinB in *Bacillus subtilis*. J Bacteriol. 177(1):166–175.

Overkamp W, Beilharz K, Detert Oude Weme R, Solopova A, Karsens H, Kovacs AT, Kok J, Kuipers OP, Veening J-W. 2013. Benchmarking Various Green Fluorescent Protein Variants in *Bacillus subtilis*, *Streptococcus pneumoniae*, and *Lactococcus lactis* for Live Cell Imaging. Appl Environ Microbiol. 79(20):6481–6490.

Shields RC, Kaspar JR, Lee K, Underhill SAM, Burne RA. 2019. Fluorescence tools adapted for real-time monitoring of the behaviors of *Streptococcus* species. Appl Environ Microbiol. 85(15):e00620-19.

Subach OM, Cranfill PJ, Davidson MW, Verkhusha V V. 2011. An enhanced monomeric blue fluorescent protein with the high chemical stability of the chromophore. PLoS One. 6(12).
